# Observations on Organic Affections of the Heart

**Published:** 1831-06

**Authors:** R. Wade

**Affiliations:** Member of the Royal College of Surgeons, and Lecturer on Morbid Anatomy, &c.


### ORGANIC AFFECTIONS OF THE HEART.

*Observations on Organic Affections of the Heart.* By R. Wade, Esq., Member of the Royal College of Surgeons, and Lecturer on Morbid Anatomy, &c.

(Concluded from page 390.)

### APPENDIX.

The following cases, selected from many similar ones, (with the exception of Nos. vi. and vii., which are the only examples of gangrene of the valve, and extensive adipose deposit under the internal arterial tunic, which have occurred to me,) are sufficient to shew the usually complicated nature of organic affections of the heart, and will be found to illustrate many of the preceding observations. On the whole, it is evident that the organs most subject to inflammation, or congestion, in these diseases, are the lungs, the liver, the mucous membrane of the stomach and small intestines, the skin, the kidneys, and the spleen.

Case I. *Pericarditis; active Dilatation of the Right Ventricle, and Contraction of the Left Auriculo-ventricular Orifice, &c.*

Mrs. Jackson, thirty-three years of age, residing at No. 14, Phœnix street, Soho; on my first visit, (June 12th, 1828,) informed me that her complaint had been of three years' duration, having commenced with slight palpitations and an uneasy sensation in the scrobiculus cordis, occasionally amounting to pain, which extended to the back and left shoulder. The palpitations and pain gradually increased, accompanied by more or less dyspnœa, especially on any exertion; a short and dry cough, frequently very troublesome; in a few months, hæmoptysis occurred, and the attacks of pulmonary hemorrhage were repeated regularly about the period of menstruation, which discharge was deficient, until within three months of the fatal termination of the disease, when they both ceased.

No sleep can be obtained when the patient lies on her back or side, and she is obliged to be raised up in her bed by pillows during the night; her sleep is often disturbed by frightful dreams; she frequently awakes suddenly with a sense of suffocation and extreme oppression in the chest: the pulse is seventy-eight, full, soft, and regular; the palpitations are described as having lately much decreased in strength; they are now, however, very distinct; occasional pains are experienced in the chest, which usually last but a few minutes; the countenance is very anxious, slightly œdematous, and the cheeks and lips are of a dusky purple; the tongue is thinly coated with a white fur; flatulence, which occurs soon after taking food of any kind, very distressing; the bowels are rather constipated, and the urine, of which there is apparently no deficiency, deposits a copious whitish sediment; the cough is seldom attended with expectoration. The action of the heart is felt distinctly, but not strongly in the erect, but cannot be distinguished in the recumbent, position.

Diuretics and purgatives were the remedies chiefly employed, and for some time they afforded great relief; the disease, however, gradually gained ground, and, on the 15th of April, 1829, the following symptoms were noted: Pulse ninety-two, small, soft, and regular; the heart's action indistinguishable in the regio cordis; the tongue moist, and slightly furred; the cheeks and lips of a deep purple, and the countenance evincing great anxiety; no pain in the chest. A fit has lately occurred once or twice during the night; the patient awakes suddenly with a sense of suffocation, cough, and giddiness; she feels "as if her head were confined in a tight place;" insensibility and convulsions supervene, which usually last for nearly a quarter of an hour. She sleeps generally in a chair, leaning forward with her elbows on her knees, and her head resting upon her hands. A fluttering is often felt at the scrobiculus cordis; the dyspnœa is extreme; the alvine evacuations are dark and offensive, and the urine very deficient; the whole surface of the body is anasarcous. The disease increased rapidly, and, two days before its fatal termination, which took place on the 23d of May, the labia became distended with serum, the inferior extremities having, for several days previously, been so excessively enlarged by the effusion, as to have occasioned ulcerations in two or three places in the legs, which latterly assumed a gangrenous aspect.

*Post-mortem Examination.* In the chest, which was well formed, the following appearances were observed: complete adhesion of the pleura pulmonalis to the pleura costalis on the right side, and partial on the left; the lungs were much consolidated in many parts, these being of a deep red colour; in others, they had very little appearance of disease. The pericardium had contracted adhesions to the pleura pulmonalis; it was very much distended, and, when divided, more than half a pint of serum escaped; its internal surface was much blanched, and, on the cardiac portion of the membrane, a considerable quantity of a dirty, yellowish lymph had been partially deposited. The apex cordis was of a bright crimson, which appearance pervaded, in small patches, other parts of the surface of the heart; the redness arising from the increased vascularity of the cellular tissue immediately under the membrane. The parietes of the left ventricle of the heart were of their usual thickness, and those of the right increased to the size of the left. All the cavities of the heart were quite empty; the large veins were distended with blood, which was very thin, and had not coagulated; the bicuspid valve was of a hard cartilaginous structure, and the left auriculo-ventricular opening so much contracted, that a small-sized quill could scarcely be passed through it; the tricuspid and other valves were free from disease; the heart was of a pale yellowish-brown colour and loose texture. The abdominal viscera, with the exception of the liver, which was much enlarged and hardened, were generally healthy.

### II. *Hypertrophy of the Left Ventricle, Hydrothorax, &c.*

Mr. ——, thirty-six years of age, stout and well formed, has been accustomed to drink freely of spirit. I saw this man occasionally during the last eighteen months of his life. He had been subject, at times, for years, to palpitations and dyspnœa, which gradually increased in frequency, especially on exertion; his pulse was seldom less than ninety, and usually above one hundred, hard and jarring; the countenance very pale, and lips of a leaden colour; the tongue preternaturally red, and furred in its centre. The patient suffered much from pains in the head and paroxysms of tic douloureux in the face, also from neuralgia affecting the sciatic nerve. About ten weeks before his death, he was seized with acute inflammation of the lungs and pleura, quickly followed by hœmoptysis. Latterly, the recumbent position could only be borne for a very short time, and an attempt to lie on the left side produced a painful sense of suffocation; respiration was very short and anxious; the cough, which had been very distressing from the commencement of the inflammation, much increased, and was attended with expectoration of a very adhesive muco-purulent matter, tinged with blood; the tongue was of a beefy redness, and coated with a thin dry varnish; pulse 130, small, hard, and vibrating; considerable difficulty in deglutition. The patient only lived two days after these symptoms were observed. No vomiting occurred during the disease, but, during his last illness, great disinclination to take nourishment of any kind was evinced.

*Post-mortem Examination.* Thorax: More than two quarts of sero-purulent fluid in the right thoracic cavity; the pleura was much thickened, and of a ragged appearance, from effused lymph; the lung was compressed against the spine and ribs, and did not appear to be larger than the palm of the hand: it had a dark chocolate colour and rotten texture. No fluid, nor adhesions, were observed in the left side of the chest; the lung had not entirely lost its crepitus, it was gorged with nearly black blood, and very soft; the bronchi were filled with a frothy bloody serum. The left ventricle of the heart was twice its healthy size, from hypertrophy; the right ventricle was exceedingly thin, not more than one eighth of an inch in thickness; the valves on both sides were healthy. The external surface of the heart had, in several places, patches of coagulable lymph. The mucous membrane of the stomach was of a dark reddish-brown colour, with very vascular spots; it was soft and considerably thickened; the lining membrane of the small intestines was also generally inflamed. The liver was much enlarged, and cut like cartilage.

### III. *Carditis, &c.*

Mrs. Hand, forty-four years of age, informed me that she had experienced constantly, for the last twelve months preceding her death, a sense of coldness and occasional pain in the præcordia, with an indescribable oppression in the chest; she had frequent fits of coughing, with very scanty mucous expectoration; the slightest exertion produced the most distressing dyspnœa, and occasionally syncope. I first saw this woman three days previous to her dissolution, when she complained of extreme difficulty of breathing, and coldness in the region of the heart; she had frequent rigors, which were not succeeded by heat of skin; her pulse was small, and about ninety. A deep inspiration occasioned no pain, but, on pressing the epigastric region, considerable uneasiness was felt, which part soon became so painful as to induce me, two days after my first visit, to have the patient cupped to f ℥viij. She was cupped at three o'clock in the afternoon, and expressed herself much relieved by the loss of blood: the extremities, however, soon became cold, and at ten o'clock dissolution took place, seven hours after the cupping: previous to which abstraction of blood, there were no symptoms which led me to expect so sudden and fatal a termination of the disease.

*Sectio cadaveris.* On opening the thorax, about an ounce and a half of serum was observed in the left side of the chest. The lungs were filled with bloody serum; the heart was small in proportion to the other parts of the body, it was remarkably pale, and of a very flabby texture, and weighed nine ounces: on the apex cordis was a patch or two of lymph, and fat had been freely deposited on the external surface of the heart; its internal membrane was of a deep crimson colour, as was also that of the aorta, to the extent of three or four inches, which appearances could not have been caused by transudation after death, as both sides of the heart were quite empty; the mitral and aortal valves thickened, and of a dark red colour; lymph had been deposited between some of the carneæ columnæ. The cellular membrane of the abdomen was loaded with fat, as was also the omentum. The mucous membrane of the stomach, and of many parts of the small intestines, was of a dark chocolate hue, and considerably thickened. The spleen felt like a bag of fluid, and, after its peritoneal coat had been cut, its contents oozed out very freely, and, except being of a darker colour and very offensive odour, had much resemblance to the secretion often observed in the mucous membrane of the stomach and intestines in a high degree of inflammation, usually compared to coffee grounds. The liver and kidneys were enlarged, of a dark red colour, and of a very soft texture.

### IV. *Passive Dilatation of the Left Ventricle, and both Active and Passive of the Right.*

Mr. Mead, aged forty, a well-formed muscular man; has been a hackney coachman during the last fourteen years. December 5th, 1825: the patient states that he was attacked, seven weeks ago, with a sharp pain in his left side, cough, great difficulty of breathing, and strong palpitations; he attributes his illness to his having been long exposed to cold and damp. The symptoms have continued, more or less severe, to the present time: the slightest exertion causes dyspnœa and strong palpitations; the countenance is anxious, and has a pale leaden aspect; the lower extremities are anasarcous. The man obtains the greatest ease by sitting up in bed with his elbows on his knees; he cannot bear the recumbent position for many minutes, when he can only lie on his right side; the cough is very troublesome, and is attended with expectoration of a viscid mucus, streaked with blood; the pulse is hard, full, and jarring, varying from ninety-five to one hundred, and the heart's action very laboured; percussion affords a very dull sound. The attendants assert that, at times, when the room is very still, they can hear the beating of the heart, like the ticking of a watch. The patient often awakes suddenly with palpitations and most distressing dyspnœa, and expresses great horror of suffocation; the alarm depicted on the countenance at these times is beyond description. The stools are dark and offensive; the stomach has been very irritable throughout the disease, and will now scarcely retain any thing; the chief pain is referred to the scrobiculus cordis, extending to the back and left shoulder.

The symptoms continued much the same until the patient's death, which took place on the 21st of December, about ten weeks from the commencement of the attack. A few hours before his dissolution, he awoke with violent palpitation, and expectorated about a teacupful of bloody mucus. The man had been a free drinker of ardent spirits. Depletion was adopted to a considerable extent, but afforded only very transient relief.

*Sectio cadaveris.* The right thoracic cavity contained a pint of serum, and the pericardium an ounce; the heart was twice its usual size; the pulmonary ventricle was much enlarged by passive dilatation; the tricuspid and the valves of the pulmonary artery were quite healthy; the parietes of the left ventricle were double their usual thickness, its cavity was dilated, but not so much as the right; the mitral and aortal valves were healthy; the internal coat of the aorta was studded, at its commencement, with spots of ossific matter. The lungs were gorged with bloody serum, and the bronchial tubes filled with frothy mucus. The liver and spleen were of a soft spongy structure. The mucous membrane of the stomach, and different parts of the intestines, had patches of chronic inflammation.

### V. *Pericarditis, with Inflammation of the Internal Membrane of the Heart and Valves.*

Mr. Juno, a stout man, forty years of age, was first attacked with dyspnœa and palpitations a year and a half ago, which gradually subsided, and he remained apparently quite well, until within six weeks of the present time, when the dyspnœa and palpitations returned. He now complains of constant pain at the scrobiculus cordis, extending to the left shoulder and elbow, and occasionally as far as the wrist: he compares the pain to "the gnawing of a dog," and has a sharp pain in the throat, as if some one were cutting it; he has severe fits of coughing. The slightest exertion brings on intense suffering, which begins with a fluttering sensation in the regio cordis: at these times, the forehead is bedewed with perspiration; the pulse is small, quick, and jarring. The symptoms gradually increased, when, about three months from the commencement of the present attack of the disease, he was bled to f℥xx. during one of the paroxysms of dyspnœa, &c. Much relief was obtained for a few minutes after the bleeding, but the patient complained of feeling very faint, and, twenty minutes afterwards, syncope occurred, from which he could not be recovered.

*Post-mortem Examination.* The pericardium had patches of lymph, both on its external and internal surfaces: it contained three ounces of bloody serum; the lining membrane of the right auricle was of a black colour, extending to some distance along the venæ cavæ; the tricuspid and mitral valves were also of a dark colour and disorganized texture, forming a striking contrast to the healthy appearance of the ventricles; the aortal valves were much thickened; the lining membrane of the aorta was of a deep crimson, and generally spotted with osseous spicula to its curvature. No other marks of disease were observed.

It is but fair to state, that this patient was not under my care, but I had an opportunity of seeing him frequently; and so great was the relief afforded by the abstraction of blood, that he was constantly soliciting to be bled: the great error was the taking so large a quantity at a time. The example may serve as a caution to many of the junior members of the profession, who are apt to commence practice with very Sangrado-like principles.

VI. *Active Dilatation of the Left Ventricle; Passive of the Right; and Gangrene of the Aortal Valve, &c.*

Mr. ——, a stout man, forty-two years of age, of temperate habits, had an attack of inflammation in the chest nine months before his death; the pain, which in the early stage of the disease was acute, and chiefly confined to the left side of the chest, latterly in a great degree subsided; but he has, during the complaint, been constantly subject to dyspnœa and cough; the pulse was generally rather small, quick, and jarring. The patient complained constantly of an uneasy sensation in the region of the heart, with occasional palpitations: its action was very laboured. He was tapped for ascites two months previous to his death; after which, a most harassing diarrhœa came on, which soon exhausted him, as he continued rapidly losing strength from its commencement, and very soon died.

*Sectio cadaveris.* The lungs, which adhered firmly on both sides to the pleura costalis, were loaded with dark blood and serum. The right ventricle of the heart was passively dilated to more than half its usual size, its parietes being much attenuated; the left ventricle was actively dilated, its parietes in some parts measuring an inch and three quarters. The mitral and aortal valves resembled parchment, and in one of the latter was a gangrenous spot as large as a pea, quite black, and of a disorganized texture; the other two were covered with warty excrescences, very similar to those often observed on the glans penis. No other marks of disease were evident.

VII. *Passive Dilatation of the Right Ventricle; both Active and Passive of the Left; and extensive Adipose Deposition under the Internal Arterial Tunic.*

August 25th, 1830. Stephen Miles, fifty years of age, well formed, and of temperate habits, a currier, states that he has generally enjoyed good health, but has been subject to an ulcer on the right inner ankle during the last twenty years, which has seldom been healed for any length of time; he has also experienced, for the last twelve months, after more than common exertion, some degree of dyspnœa and palpitations. Nine weeks ago, with no other premonitory symptoms than those previously mentioned, the patient awoke in the night with extreme difficulty of breathing and pain in the chest, which obliged him to sit up in bed, and he has never since been able to bear the recumbent position for any length of time. The paroxysms of pain and dyspnœa are frequently so severe as to cause him often to start up and rush across his room in a state of distraction, and so great is then the sense of suffocation, that he is anxious to have all the windows and doors open; a constant burning pain and throbbing are felt at the scrobiculus cordis, and the ensiform cartilage is forcibly elevated at every stroke of the heart. The paroxysms of extreme suffering usually last from three to four hours, when the urine, which is deficient and high-coloured, is passed involuntarily. The man was able to pursue his usual employment for a fortnight after the first paroxysm of his disease: in a month, the lower extremities became œdematous. The patient finds himself easiest when sitting with his elbows upon a table, and his head reclined forwards and sopported on his hands: the pulse was small, frequent, regular, and vibrating; respiration short and anxious; there is extreme tenderness and strong pulsation at the scrobiculus cordis; the bowels are constipated, and the fæces of a pale yellow colour; the surface of the body cold, and the lower extremities, as high as Poupart's ligament, are enormously distended with serum: a constant and most distressing flatulence is experienced, and any thing taken into the stomach causes so much pain, that it is difficult to persuade the patient to take even a little arrowroot. A short hacking cough, with but little expectoration, has been present from the commencement of the disease; the tongue is very red; occasional nausea, but no vomiting is experienced. Dissolution took place suddenly two days after my first having seen the patient.

*Sectio cadaveris.* On examination of the body four hours after death, the muscles were observed to be generally small and flabby; the deposition of adeps was unusually great: the inferior extremities were very much distended with serum, and on the right inner ankle was an ulcer, surrounded, to some extent, by a purple discoloration of the skin. The heart was excessively enlarged; the pericardium had its vessels, in several parts, minutely injected, and contained an ounce of serum; the right auricle and ventricle were passively enlarged to twice their usual size; the tricuspid valve had lost its transparency; the left ventricle was found to be both actively and passively dilated; the left cavities of the heart were empty, but the right distended with coagulated blood. The commencement of the aorta and pulmonary arteries was very much dilated. The internal membrane of the arteries was extensively diseased, in some few places by the atheromatous deposit, but chiefly from a deposition of fat immediately beneath it: the disease extended beyond the bifurcation of the iliacs; the innominata, subclavian, and carotid arteries were also very generally studded with a similar adipose matter. The brachial artery was examined at the inner edge of the biceps, and found to be in a healthy state, as were also the lungs and pleuræ. The mucous membrane of the stomach was generally of a dark red colour, much softened, and had an ecchymosed appearance, resulting from a minute injection of vessels, (and not from extravasation, as is often supposed,) as was evident when examining it with a microscope. The mucous lining of the small intestines was loaded with blood of a dark venous colour, and much softened. The liver was enlarged and hardened: the pulsation at the scrobiculus cordis was occasioned by the edge of the liver lifting up the ensiform cartilage at every stroke of the enlarged heart.

### VIII. *Adherent Pericardium, Inflammation of the Lining Membrane of the Heart, &c., and Cancer Uteri.*

Mrs. Harriss, thirty-nine years of age, states that, about five years ago, she experienced some degree of uneasiness in her back and loins, which soon increased to pain: the menstrual discharge became irregular about this period, and she was constantly affected with leucorrhœa, which discharge, after some length of time, became very offensive and tinged with blood. The symptoms of the disease gradually increased, and, for the last six months of her life, the urine passed involuntarily: she was also affected with a slight dry cough. The pulse was latterly very small, and the pain in the loins extremely severe. The patient could lie either on her back or sides. She complained of a slight degree of dyspnœa, on any exertion. Profuse hemorrhage took place, per vaginam, a few days before dissolution; which fatal termination of the disease occurred rather more than five years from its commencement.

*Sectio cadaveris.* Thorax: Adhesion of the pleura costalis to the pleura pulmonalis on the right side. The lung was loaded with bloody serum, and the bronchi with frothy mucus. The pericardium adhered completely to the heart, and nearly the whole of the membrane covering the right ventricle, to the diaphragm. The lining membrane of the left ventricle was thickened, and of a straw colour, from matter effused beneath it; and its parietes were of a flabby texture, and very pale; the mitral and aortal valves were thickened; the right ventricle presented a remarkable contrast to the left, as both its lining membrane and muscular fibres were of a healthy appearance. All the cavities of the heart were empty. No disease was observed in the left lung.

Abdomen: The fundus uteri had a hard cartilaginous structure: the whole of its cervix had been destroyed by ulceration, as had also the inferior portion of the bladder, which, with the uterus, formed but one cavity; the left ovarium adhered to the sigmoid flexure of the colon; the ovarium were much enlarged, and schirrous; a small cyst, containing a gelatinous fluid, was attached to the right. The mucous membrane of the small intestines was generally inflamed, and very pulpy. The rectum and colon were free from disease.

*Active Dilatation of the Right Ventricle; Hydrothorax, &c.*

Hannah Woodcock, twenty-six years of age, with mal-formed chest, from curved spine, states that, about three years ago, she was attacked with severe pain in the right side, attended with fever, with constant cough and difficulty of breathing; which symptoms were completely relieved by bleeding. She remained well for twelve months, at which time she had another attack of inflammation in the chest, which subsided without medical aid. The disease again returned about fourteen months from the present period, when the lower extremities soon became œdematous: she has suffered constantly ever since with pain in the chest and most distressing dyspnœa. The pain is now chiefly confined to the region of the heart, but occasionally extends to the left shoulder, and along the arm as far as the wrist. The patient complains very much of an uneasy feeling in the epigastrium, as if "something turned up," (to use her own expression:) occasionally, severe pain in the head and vertigo are experienced: the lower extremities are very much distended, and fluctuation can be distinctly felt in the abdomen. The appetite is good, but great oppression is produced after taking food. The recumbent position cannot be borne; the patient generally kneels during the night, with her head and elbows upon a table, and cannot procure sleep in any other position. The pulse is 106, and hard; countenance anxious and bloated; the lips and tongue of a purple colour; the cough is attended with a scanty expectoration of frothy mucus, which, in a morning, is tinged with blood; the urine is very deficient, and deposits a copious red sediment; the bowels were usually constipated. Diuretics and purgatives afforded the greatest relief, and for a time appeared completely to have removed the effusion, which, however, after a short time, returned, and the disease soon terminated fatally.

*Sectio cadaveris.* Thorax: Eight ounces of fluid had been effused in the right cavity of the chest, and, in the left, the pleura pulmonalis adhered to the pleura costalis; the lungs were in some parts much consolidated, and in others surcharged with bloody serum. The apex cordis was fixed to the pericardium, by a broad and firm band of coagulable lymph; the right ventricle of the heart was actively dilated, being much thicker than the left, and of a hardened texture, nearly cartilaginous in places; the columnæ carneæ were remarkably irregular, hard, and ragged, from effused lymph, which had agglutinated them together. The left ventricle was healthy in appearance and structure.

Abdomen: In this cavity, about four quarts of fluid were observed; the liver much enlarged, and gorged with very dark-coloured blood. The mucous membrane of the intestines was very red, thickened, and pulpy; the kidneys were nearly in contact with the diaphragm, and lying horizontally on the shelf formed by the curved spine.

X. *Hypertrophy of the Left Ventricle, and Disease of the Aortal and Mitral Valves.*

A young man, seventeen years of age, had been long subject to dyspnœa, to strong palpitations, and constant dry, hacking cough: his countenance was very anxious; the heart's action was felt powerfully over the chest, but the pulse at the wrist was quick, exceedingly small, and jarring: occasional pain was felt in the region of the heart. He had been quite as well as usual, being able to attend to his work, as a tailor, until the day of his death, when, on his return home, after having walked nearly a mile, he complained of giddiness and dimness of sight: convulsions quickly followed, and in two hours the case terminated fatally.

*Post-mortem Examination.* The pleura pulmonalis adhered firmly to the pleura costalis on the right side; no adhesions on the left; the lungs were gorged with black blood. The parietes of the left ventricle of the heart were much thickened, and its cavity very much contracted; the mitral and aortal valves were inflamed and ossified; the right ventricle was exceedingly thin. No other diseased appearances were observed, with the exception of a congestive state of the cerebral veins.

68, *Dean street, Soho; March* 1831.

---

